# Effect of Local Laser Therapy on Plantar Fasciitis: A Meta-Analysis

**DOI:** 10.3390/jcm15031307

**Published:** 2026-02-06

**Authors:** Mercedes Ortiz-Romero, Gabriel Gijón-Noguerón, Pablo Rodríguez de Vera-Gómez, David Rodríguez de Vera-Gómez, Nerea Escribano-Rodríguez, Luis María Gordillo-Fernández

**Affiliations:** 1 Department of Podiatry, Faculty of Nursing, Physiotherapy and Podiatry, University of Seville, 41004 Seville, Spain; lgordillo@us.es; 2 Biomedical Research Institute (IbiS), 41013 Seville, Spain; 3 Department of Nursing and Podiatry, Faculty of Health Sciences, University of Malaga, Arquitecto Francisco Peñalosa 3, Ampliación de Campus de Teatinos, 29071 Malaga, Spain; 4 IBIMA Plataforma BIONAND, 29010 Malaga, Spain; 5 Endocrinology and Nutrition Service, Virgen Macarena University Hospital, 41009 Seville, Spain; 6 Faculty of Podiatry, University of Seville, 41004 Seville, Spain

**Keywords:** plantar fasciitis, laser, photobiomodulation, LLLT, HILT, meta-analysis

## Abstract

**Background/Objectives:** Plantar fasciitis (PF) is a leading cause of heel pain and functional impairment in adults. Laser therapy, in its low-intensity laser therapy (LLLT), high-intensity laser therapy (HILT), and photobiomodulation (PBMT) modalities, has been proposed as a non-invasive alternative, although its clinical effectiveness remains a subject of debate. The aim of this systematic review and meta-analysis was to evaluate the efficacy of laser therapy in reducing pain, improving function, and modifying fascial thickness in patients with PF. **Methods**: A comprehensive search was conducted in PubMed, Embase, and PEDro (last search: August 2025). Randomized controlled trials comparing laser therapies versus placebo or alternative physical interventions were included. Two reviewers performed study selection, data extraction, and risk of bias assessment using the PEDro scale. Random-effects meta-analyses were performed for pain (VAS), heel tenderness (HTI), function (FFI, AOFAS, ASQoL, SF-36), and fascial thickness, expressing effects as standardized mean differences (SMDs) or mean differences (MDs). **Results**: Thirteen trials with 784 participants were included. Laser therapy showed a significant improvement in heel tenderness (SMD = −0.40; 95% CI −0.71 to −0.09; I^2^ = 0%). No significant differences were observed in overall pain (SMD = −0.18), function (SMD = 0.20), or fascial thickness (MD = −0.18 mm). Pain and function analyses showed high heterogeneity. **Conclusions**: Laser therapy may reduce heel tenderness in plantar fasciitis, but it does not consistently improve overall pain, function, or fascial thickness. Its use should be considered as a therapeutic adjunct and not as a primary intervention. Larger trials with standardized protocols are needed.

## 1. Introduction

Plantar fasciitis (PF) is one of the most common causes of heel pain and is characterized by degenerative and inflammatory changes to the plantar fascia. This condition can lead to significant functional limitations and a marked reduction in quality of life [1]. Its prevalence is particularly high in the adult population, especially among physically active individuals, athletes, and people with overweight or obesity, making PF a frequent and clinically relevant condition in musculoskeletal medicine [1,2].

A wide range of conservative treatments is currently available for PF, including stretching exercises, foot orthoses, extracorporeal shock wave therapy (ESWT), and manual therapies. In recent years, increasing attention has been directed toward low-level laser therapy (LLLT) as a non-invasive therapeutic option [2]. LLLT involves the application of coherent light at specific wavelengths—most commonly within the infrared spectrum—to induce photobiological effects at the cellular level. These effects include enhanced mitochondrial activity and ATP synthesis, modulation of oxidative stress, and attenuation of inflammatory processes, all of which may contribute to pain relief and tissue recovery [2].

Several randomized controlled trials have investigated the effects of LLLT on pain and functional outcomes in patients with plantar fasciitis. For instance, one controlled trial compared LLLT (850 nm irradiation applied over multiple sessions) combined with exercise and orthotic support versus conventional treatment, reporting greater improvements in functional outcomes measured by the American Orthopaedic Foot & Ankle Society (AOFAS) score, as well as greater pain reduction after three months in the LLLT group [3]. Another trial compared LLLT with ESWT and found that, although both interventions led to pain improvement, LLLT did not demonstrate a significantly greater short-term analgesic effect compared with ESWT [4].

From an evidence synthesis perspective, several meta-analyses have attempted to clarify the clinical effectiveness of LLLT in plantar fasciitis. A meta-analysis by Wang et al. [1], which included six randomized controlled trials, concluded that LLLT significantly reduces pain intensity as measured by the visual analog scale (VAS) at the end of treatment, with effects maintained for at least three months [1]. Although no statistically significant improvement was observed in functional outcomes assessed using the Foot Function Index pain subscale (FFI-p), the authors suggested that the sustained pain reduction may still represent a clinically meaningful benefit [1]. A more recent and comprehensive meta-analysis by Naterstad et al. [2] analyzed randomized controlled trials involving plantar fasciitis and related lower limb tendinopathies and reported that LLLT—particularly when delivered according to the dosage recommendations of the World Association for Laser Therapy (WALT)—results in significant short- and medium-term reductions in both pain and disability [2].

Despite these encouraging findings, important limitations remain. Many of the available trials are characterized by relatively small sample sizes, substantial variability in laser parameters (including wavelength, energy dose, and treatment frequency), and a lack of long-term follow-up data [1,2,5]. Moreover, comparisons between LLLT and other physical modalities, such as ESWT or high-intensity laser therapy (HILT), have yielded inconsistent results. In a recent randomized clinical trial, no statistically significant differences in pain reduction were observed between LLLT and HILT, although patients treated with HILT reported a greater subjective perception of treatment effectiveness [6].

Despite the availability of established first-line conservative treatments for plantar fasciitis, such as stretching-based physiotherapy, orthotic interventions, and extracorporeal shock wave therapy, the clinical role of laser therapy remains unclear. While laser-based interventions are increasingly used in clinical practice, uncertainty persists regarding their comparative effectiveness and their appropriate position within current treatment algorithms. Therefore, the objective of this systematic review and meta-analysis was to evaluate the effectiveness of laser therapy in the management of plantar fasciitis, with particular focus on pain-related outcomes (including heel tenderness and overall pain), functional outcomes, and plantar fascia thickness, and to clarify the potential role of laser therapy as an adjunct to established conservative treatments.

## 2. Method

This systematic review was carried out according to a protocol registered in advance in PROSPERO under registration number CRD420261282561 and is presented in line with the 2020 Preferred Reporting Items for Systematic Reviews and Meta-Analyses guidelines [7]. The PRISMA 2020 checklist is provided as Supplementary Material.

### 2.1. Literature Search and Study Selection

Only randomized controlled trials exclusively including participants diagnosed with plantar fasciitis were eligible for inclusion; studies primarily addressing other tendinopathies were excluded.

Randomized controlled trials evaluating LLLT or HILT/PBMT in individuals with plantar fasciitis were eligible when compared with sham LLLT (placebo), alternative interventions (such as ultrasound, extracorporeal shock wave therapy, orthotics, or exercise), or no treatment. Primary outcomes included patient-reported measures of pain, functional status, and disability. No limitations were imposed on publication date or language.

A comprehensive search for eligible randomized trials was performed on 20 August 2025, using the PubMed, Embase, and Physiotherapy Evidence Database (PEDro) databases. Reference lists of relevant systematic reviews and of all included studies were also screened, and field experts were consulted to identify both published and unpublished work. Conference abstracts were excluded. The complete PubMed search strategy is provided in the online Supplementary Material.

Two reviewers (IFN and MBS) independently screened the titles and abstracts of all retrieved records. Full-text versions of potentially relevant articles were subsequently evaluated according to predefined inclusion and exclusion criteria. Discrepancies were resolved through discussion and consensus, with arbitration by a third reviewer (JJ) when required.

### 2.2. Risk of Bias Analysis

Risk of bias was independently evaluated by two reviewers (IFN and MBS) using the PEDro scale, which assigns scores ranging from 0 to 10 points. The assessment was conducted at the outcome level, because the primary outcomes were patient-reported measures of pain and disability, participant blinding was considered particularly relevant. Outcome assessor blinding was only feasible in trials using placebo or sham-controlled designs. Consequently, assessor blinding was feasible only in trials with a placebo control. Studies were categorized as having high, moderate, or low methodological quality when total PEDro scores were ≥7, 5–6, or ≤4, respectively [8]. Publication bias was assessed by visual inspection of funnel plots. Formal statistical tests for funnel plot asymmetry, such as Egger’s test, were not performed due to the small number of studies included in each meta-analysis, in accordance with methodological recommendations.

In addition to the overall PEDro scores, key risk-of-bias domains were considered qualitatively, including random sequence generation, allocation concealment, blinding of participants and assessors, completeness of outcome data, and selective outcome reporting. Most trials showed adequate randomization and baseline comparability, while blinding of therapists and assessors was limited and largely restricted to placebo-controlled studies. These domain-level considerations were taken into account when interpreting the results.

### 2.3. Data Extraction and Meta-Analysis

Although included studies used a wide range of laser parameters, both LLLT and HILT were considered under the broader framework of photobiomodulation, given their shared biological mechanisms. Pooling these modalities was intended to maximize statistical power; however, this approach inherently introduces clinical heterogeneity, which was addressed through random-effects models and is considered in the interpretation of the results.

For each included trial, the following data were required: sample size in the laser and control groups, participant demographic and clinical characteristics, intervention type and duration, and detailed parameters of laser application, including treatment site, wavelength, energy delivered per point, number of application points, mean power per point, irradiation time per point, treated area, treatment frequency, and total number of sessions. Information on outcome measurement instruments, timing of assessments, effect size estimates, and reported adverse events was also extracted.

Data extraction was conducted independently by two reviewers (IFN and MBS). One reviewer recorded the data in Excel spreadsheets, while the second reviewer cross-checked all entries for accuracy. In cases where discrepancies could not be resolved through discussion, a third reviewer (JMB) adjudicated to reach consensus. Meta-analyses were carried out using random-effects models, which assign relatively balanced weights to individual studies in the presence of statistical heterogeneity [9].

Pain-related and functional outcomes were pooled using standardized mean differences (SMD, Hedges g), based on change-from-baseline scores whenever available, to account for the use of different measurement instruments across studies. Mean differences (MD) were used only for outcomes measured on the same scale, such as plantar fascia thickness (mm) [9]. Change scores and post-intervention values were not combined within the same meta-analysis; for each outcome, the most consistently reported metric across studies was used. Pain intensity assessed with the Visual Analogue Scale (VAS) and the Numerical Rating Scale was considered comparable due to their high correlation [10]. Outcomes related to patient-reported disability were combined using the standardized mean difference (SMD), to accommodate the use of different instruments assessing related aspects of function and disability. This analysis was intended to provide a global estimate of functional impact rather than instrument-specific effects. In line with Cohen’s criteria, SMD values of 0.2, 0.5, and 0.8 were interpreted as small, moderate, and large effects, respectively [9].

Statistical heterogeneity was quantified using the I^2^ statistic [10,11]. Values of approximately 25%, 50%, and 75% were interpreted as indicating low, moderate, and high levels of heterogeneity, respectively. When standard deviations (SDs) were not directly reported, they were obtained or estimated from alternative measures of variability following a predefined hierarchy: SD, standard error (SE), 95% confidence interval (CI), *p*-values, interquartile range (IQR), median correlations, visual extraction from graphical data, an assumed correlation coefficient of 0.6, or the mean SD derived from comparable studies [12].

Studies were stratified according to laser dose in line with the World Association for Laser Therapy (WALT) recommendations, as defined in the a priori protocol [13]. WALT advises irradiation of at least two to three points within the affected tendon or fascia. In the context of plantar fasciitis, the recommended minimum dose is 2 J per point for 904 nm lasers and 4 J per point for lasers with wavelengths between 780 and 860 nm. Whenever possible, trials were categorized as using either recommended or non-recommended laser doses; studies lacking sufficient dosing information were classified as having an uncertain laser dose. Although studies were categorized according to adherence to WALT-recommended doses, formal subgroup meta-analyses were not performed due to the limited number of studies per outcome and subgroup, as well as incomplete or heterogeneous reporting of dose parameters across trials.

Two time points were predefined for analysis: the first immediately following completion of LLLT and the second corresponding to the final follow-up assessment conducted between 2 and 12 weeks after treatment. Meta-analyses were performed by IFN and MBS using Microsoft Excel 2016 and Review Manager (RevMan) version 5.3 [14,15].

All outcome data were checked and, where necessary, re-coded to ensure a consistent direction of effect, such that negative values uniformly reflected clinical improvement for pain- and function-related outcomes prior to effect size calculation.

### 2.4. Evaluating the Certainty of the Evidence

The certainty of evidence for each outcome was evaluated using the Grading of Recommendations Assessment, Development and Evaluation (GRADE) approach [14], which is widely used in systematic reviews and clinical practice guidelines. This framework considers several domains, including the applicability of the evidence to the target population, risk of bias, consistency of results across studies, precision of effect estimates, and potential publication bias. Based on this structured assessment, the certainty of evidence was classified into four levels: high, moderate, low, or very low. This process allowed for a transparent and systematic appraisal of confidence in the estimated effects for each outcome [14,15].

### 2.5. Use of Artificial Intelligence

Artificial intelligence tools were used during the preparatory phase of this study to assist in identifying recent and relevant scientific literature to be reviewed and considered for inclusion in the discussion. These tools were employed exclusively as a support for literature discovery and did not replace manual screening or critical appraisal by the authors. In addition, Google Gemini was used to assist in formatting all references according to the Vancouver citation style. Final verification of reference accuracy and formatting was performed by the authors prior to submission. Finally, LorcaEditor was used as a language-support tool to review spelling and writing quality, including the detection of incorrectly structured sentences and excessively repeated words. All suggested edits were assessed and implemented at the authors’ discretion, and the final text was approved by the authors.

### 2.6. Patient and Public Participation

Neither patients nor the public participated in the conceptualization or execution of this research.

## 3. Results

**Figure 1:**
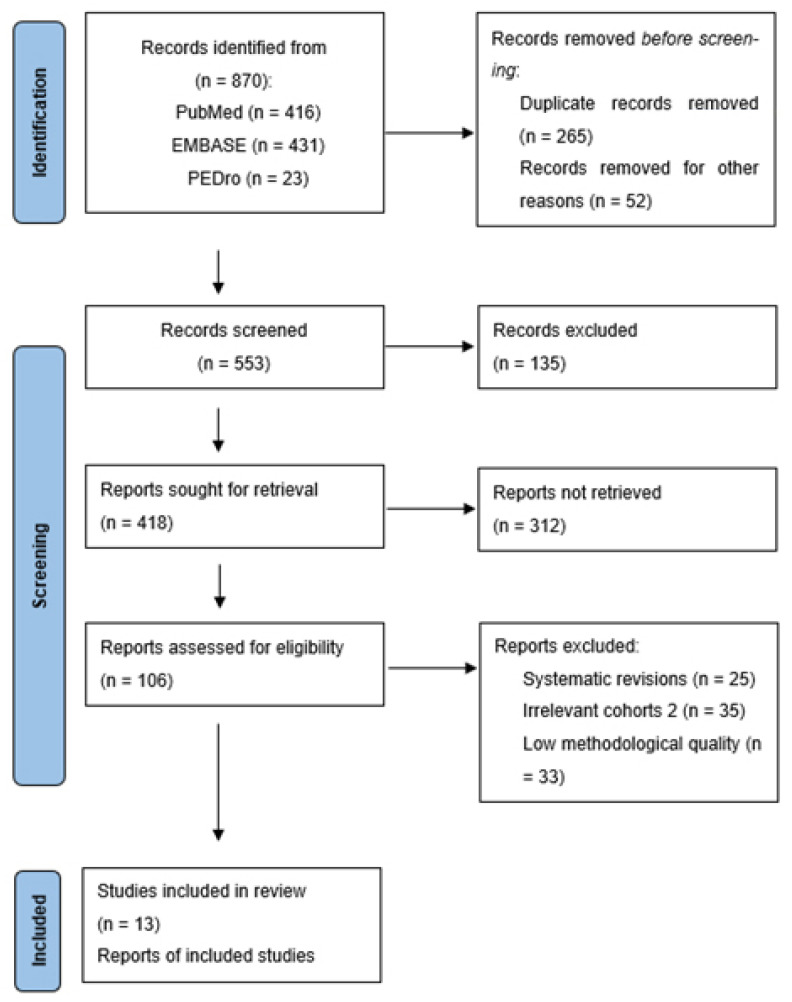
Flowchart illustrating the process of identifying trials.

A total of 13 clinical trials with 784 participants evaluating the efficacy of laser therapies in patients diagnosed with plantar fasciitis were identified and included in the meta-analysis (Figure 1). These comprised 13 randomized clinical trials involving a total of 784 participants that evaluated the efficacy of laser-based interventions for plantar fasciitis (Table 1).

Low-level laser therapy (LLLT) was investigated in eight trials, high-intensity laser therapy (HILT) in five trials, and photobiomodulation therapy (PBMT) in one trial. In several studies, laser therapy was combined with co-interventions such as exercise programs, foot orthoses, or ultrasound therapy. Comparator conditions included placebo or sham laser therapy in four trials, other physical interventions in seven trials, and usual care or adjunctive treatment in five trials. Across the included studies, participants had a mean age of 43.6 years (range: <18 to 54.5 years) and a mean baseline pain intensity of 64.2 mm on the visual analog scale (VAS).

Several studies reported improvements in pain and functional outcomes following laser therapy. For instance, Macias et al. [15] observed a mean reduction in VAS pain of −29.6 ± 24.9 points at 8 weeks in the LLLT group, compared with −5.4 ± 16.0 points in the placebo group (*p* < 0.001). Comparative trials evaluating high-intensity laser therapy (HILT) versus LLLT or extracorporeal shock wave therapy (ESWT) reported greater reductions in pain and improvements in functional outcomes and quality of life in some analyses [6,16,25].

Studies in which laser therapy was combined with exercise programs or foot orthoses, such as those by Akkurt et al. [17] and Gökçe et al. [24], demonstrated greater improvements in pain, disability, and functional measures compared with control interventions. In trials comparing LLLT with ultrasound therapy or ESWT, reductions in plantar fascia thickness and functional improvements were observed in both intervention groups, although statistically significant differences favored one treatment over the other depending on the specific outcome assessed [18,19,20,21].

In follow-up assessments, reductions in pain and improvements in function were maintained for up to 13–26 weeks after photobiomodulation therapy (PBMT) and laser-based combination interventions, with clinically meaningful changes compared with usual care. No serious adverse events were reported in the included trials; however, adverse event reporting was limited or absent in several studies. None of the included trials explicitly reported receiving funding from the laser industry.

Table 2 summarizes the application characteristics of low-level laser therapy (LLLT) and high-intensity laser therapy (HILT), including photobiomodulation therapy (PBMT), across the included studies. The wavelengths used ranged from 635 nm to 1064 nm for LLLT and HILT, with some trials employing combined wavelengths (e.g., 810 and 980 nm). Output power varied substantially, from 17 mW in conventional LLLT protocols to 30 W in high-power PBMT applications. Exposure time per treatment point ranged from 12 s to 720 s, while delivered energy per point or per treated area ranged from 2 J/cm^2^ to a total energy of up to 3000 J per session in HILT protocols, depending on the specific treatment design.

The number of treated points or scanned areas ranged from 1 to 5 per session, and the total number of treatment sessions varied from 6 to 15, typically delivered over 2–3 weeks at a frequency of 2–5 sessions per week. Where applicable, laser dosages were compared with the recommendations of the World Association for Laser Therapy (WALT). Studies were categorized according to whether the reported laser dose was consistent with WALT recommendations, non-recommended, or uncertain, in order to descriptively characterize dosing heterogeneity across trials.

Overall, the included studies demonstrated substantial heterogeneity in laser treatment parameters, including wavelength, output power, exposure time, energy delivery, and treatment frequency. This variability reflects the wide range of protocols currently used in both clinical practice and research on plantar fasciitis and related tendinopathies.

The very high heterogeneity observed in this analysis reflects substantial variability in effect estimates across studies. For example, Macias et al. [15] reported a large effect in favor of laser therapy, whereas Gökçe et al. [24] reported a large effect favoring the control intervention. These discrepancies may be partly explained by differences in laser parameters, comparator treatments, baseline pain severity, and study design. This level of heterogeneity indicates that the pooled effect estimate for pain should be interpreted with caution. Extreme effect estimates observed in individual studies may also contribute to the high heterogeneity, despite harmonization of outcome direction across trials.

### 3.1. Risk of Bias

Of the 13 included trials, 10 were rated as having high methodological quality, while the remaining 3 were classified as moderate quality (Table 3). All studies reported adequate randomization procedures. Allocation concealment was judged to be sufficient in 8 trials (62%). Baseline comparability between intervention and control groups was confirmed in 11 studies (85%).

Participant blinding was achieved in 7 trials (54%). Therapist blinding was reported in 4 trials (31%), all of which employed a placebo-controlled design. Outcome assessors were blinded in 5 studies (38%), also exclusively in placebo-controlled trials. Outcome data were available for more than 85% of participants in 10 trials (77%), and an intention-to-treat analysis was conducted in 7 studies (54%). All included trials performed statistical comparisons between groups, and point estimates together with measures of outcome variability were reported in 12 trial reports (92%).

### 3.2. GRADE: Certainty of the Evidence

Table 4 summarizes the certainty of evidence for the thirteen included randomized controlled trials according to the GRADE framework. Overall, most studies were judged to have a moderate risk of bias, primarily due to limitations in blinding of participants, therapists, and outcome assessors. These limitations were especially evident in trials comparing laser therapy with active interventions rather than with sham or placebo controls.

Inconsistency varied according to the outcome evaluated. It was low for heel tenderness (HTI) and plantar fascia thickness, reflecting minimal statistical heterogeneity, but ranged from moderate to high for pain and functional outcomes. This variability was driven by substantial heterogeneity (I^2^ > 50–90%) and differences across studies in laser modality, dosimetric parameters, treatment duration, and comparator interventions. Indirectness was consistently low, as the study populations, interventions, and outcome measures were directly applicable to patients with plantar fasciitis. Imprecision was generally moderate because of relatively small sample sizes and wide confidence intervals, and high in studies with very small samples, thereby reducing confidence in the precision of their effect estimates. Publication bias was considered unlikely, as no evidence of selective reporting was identified.

Overall, the certainty of evidence was rated as moderate for most outcomes, indicating that the true effect is likely close to the estimated effect, although clinically important differences cannot be definitively excluded. Outcomes characterized by marked imprecision were downgraded to low certainty. Taken together, the evidence supports a consistent beneficial effect of laser therapy on heel tenderness, whereas its effects on overall pain, physical function, and plantar fascia thickness remain inconsistent. These findings reinforce the role of laser therapy as an adjunctive treatment rather than as a standalone intervention for PF.

### 3.3. Meta-Analysis

#### 3.3.1. Pain (VAS/VAS, Baseline–Final Δ)

**Figure 2:**
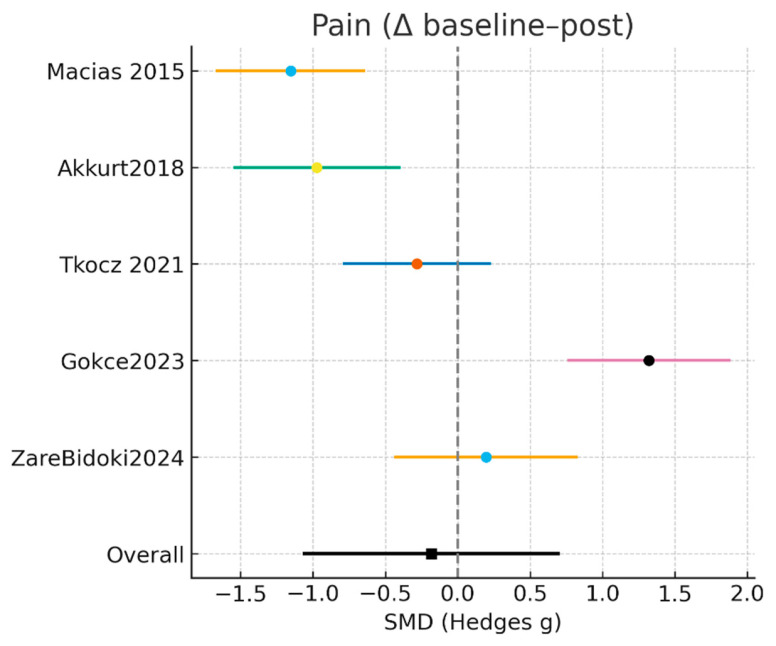
Results of the meta-analysis for pain [15,17,22,24,25].

Five studies, including a total of 141 participants in the laser intervention groups and 138 in the control groups, evaluated pain intensity using self-reported measures assessed before and after the intervention. All studies employed continuous pain scales, enabling the calculation of standardized mean differences (SMDs) based on pre- to post-treatment changes. The random-effects meta-analysis showed a non-significant pooled effect in favor of laser therapy (SMD = −0.18; 95% CI: −1.06 to 0.69; Z = −0.41; *p* = 0.68) (Figure 2). Statistical heterogeneity was very high (Tau^2^ = 0.920; χ^2^ = 51.14, df = 4; I^2^ = 92.2%), indicating substantial variability across studies in participant characteristics, laser modality, treatment duration, and pain assessment methods.

At the individual study level, Macias et al. [15] reported the largest pain reduction favoring laser therapy (SMD = −1.15; 95% CI: −1.66 to −0.65), followed by Akkurt et al. [17], which also demonstrated a clinically relevant effect (SMD = −0.97; 95% CI: −1.54 to −0.40). In contrast, Tkocz et al. [22] observed a small and non-significant effect (SMD = −0.28; 95% CI: −0.78 to 0.22). Two studies favored the comparator intervention: Gökçe et al. [24] reported a large effect in favor of the control group (SMD = 1.32; 95% CI: 0.76 to 1.87), whereas Zare Bidoki et al. [25] found a small, non-significant effect in the same direction (SMD = 0.19; 95% CI: −0.43 to 0.82).

Overall, these findings indicate that laser therapy does not consistently provide superior pain reduction compared with comparator interventions in patients with plantar fasciitis. Nevertheless, the pronounced between-study heterogeneity suggests that treatment-related factors—such as laser modality (LLLT vs. HILT), applied dosage and intensity, baseline pain severity, and patient-specific clinical characteristics—may substantially influence the magnitude and direction of the observed effects.

The results of the meta-analysis for pain intensity (baseline–final change) are presented in Table 5.

#### 3.3.2. Function/Disability (FFI Total, ASQoL, SF-36, Δ)

**Figure 3:**
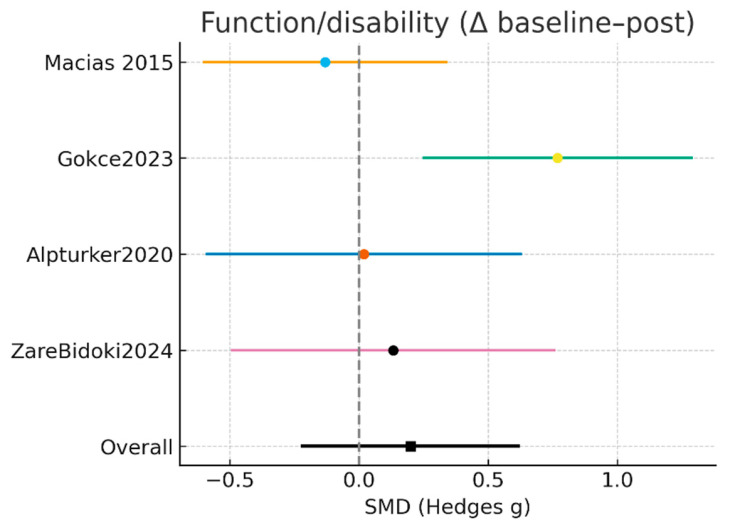
Meta-analysis results for Function/Disability [15,20,24,25].

Four randomized controlled trials, comprising a total of 106 participants in the laser intervention groups and 101 in the control groups, evaluated changes in physical function using validated outcome measures, including the Foot Function Index (FFI), the Ankylosing Spondylitis Quality of Life questionnaire (ASQoL), and the Short Form–36 Health Survey (SF-36). Pre- to post-intervention changes were analyzed to estimate the effect of laser therapy on functional outcomes compared with conventional or alternative treatments. The random-effects meta-analysis did not demonstrate a statistically significant effect of laser therapy on physical function (SMD = 0.20; 95% CI: −0.22 to 0.62; Z = 0.94; *p* = 0.35). Between-study heterogeneity was moderate (Tau^2^ = 0.102; χ^2^ = 6.92; df = 3; I^2^ = 56.6%), indicating variability in outcome measures, intervention protocols, and baseline participant characteristics (Figure 3).

At the individual study level, Macias et al. [15] reported a small, non-significant effect favoring laser therapy (SMD = −0.13; 95% CI: −0.60 to 0.34). Comparable null effects were observed in the trials by Alpturker et al. [20] (SMD = 0.02; 95% CI: −0.59 to 0.63) and Zare Bidoki et al. [25] (SMD = 0.13; 95% CI: −0.49 to 0.76). In contrast, Gökçe et al. [24] demonstrated a statistically significant effect favoring the control intervention (SMD = 0.77; 95% CI: 0.25 to 1.29), indicating greater functional improvement in participants not receiving laser therapy.

Overall, the available evidence suggests that laser therapy does not confer a superior functional benefit compared with comparator interventions in patients with plantar fasciitis. The presence of moderate heterogeneity and methodological variability across studies underscores the need for well-designed randomized trials with larger sample sizes and standardized functional outcome measures to enable more definitive conclusions.

Given the heterogeneity of functional instruments included, these results represent an overall estimate of functional change and should not be interpreted as reflecting improvements in specific functional domains.

The results of the meta-analysis for functional outcomes (baseline–final change) are shown in Table 6.

#### 3.3.3. Plantar Fascia Thickness (Δ mm, Baseline–Final)

**Figure 4:**
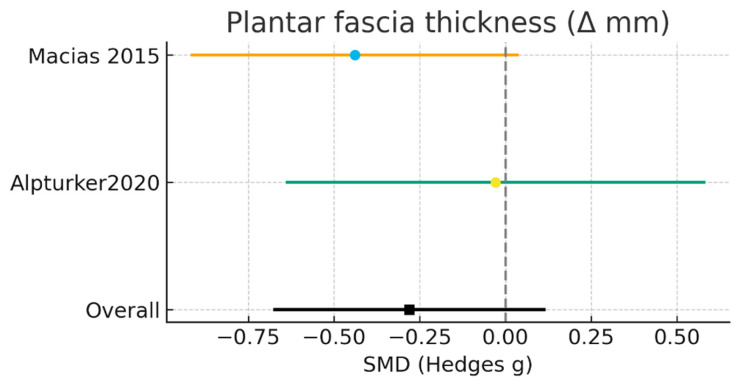
Meta-analysis results for plantar fascia thickness [15,20].

Three randomized controlled trials, comprising a total of 87 participants in the laser intervention groups and 82 in the control groups, evaluated changes in plantar fascia thickness following treatment completion. All trials employed standardized ultrasound measurements, allowing direct comparison of post-intervention fascial thickness between groups. The random-effects meta-analysis did not reveal a statistically significant difference between laser therapy and control interventions (MD = −0.18; 95% CI: −0.67 to 0.11; Z = −1.40; *p* = 0.16). Between-study heterogeneity was very low (I^2^ = 8.4%; Tau^2^ = 0.007; χ^2^ = 1.09; df = 1), indicating a high degree of consistency across studies despite differences in ultrasound equipment and laser application protocols (Figure 4).

Individual study analyses demonstrated small and non-significant effects in all three trials. Macias et al. [15] reported a minimal between-group difference (MD = −0.20; 95% CI: −0.74 to 0.34), while Rubella et al. [19], which contributed the greatest statistical weight to the pooled estimate, also observed a small effect (MD = −0.18; 95% CI: −0.37 to 0.01). Similarly, Alpturker et al. [20] found no clinically meaningful difference between interventions (MD = −0.09; 95% CI: −0.42 to 0.24).

Overall, the available evidence indicates that laser therapy does not produce an additional reduction in plantar fascia thickness compared with comparator treatments. Although the findings are consistent, the limited number of studies and heterogeneity in laser dosimetry and application protocols underscore the need for further well-designed trials with standardized methodologies and larger sample sizes to allow more definitive conclusions.

Post-treatment pain intensity outcomes are summarized in Table 7.

#### 3.3.4. Pain Measured by FFI (Pain Subscale, Δ)

**Figure 5:**
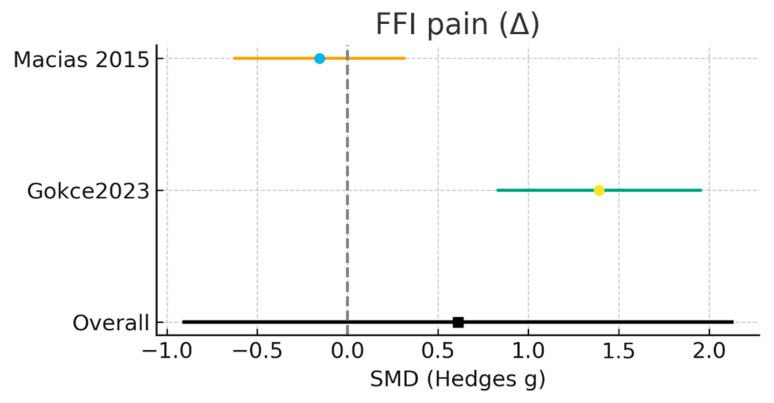
Results of the meta-analysis of pain measured by the FFI [15,24].

Two randomized controlled trials, including a total of 67 participants in the laser intervention groups and 62 in the control groups, evaluated pain outcomes using the pain subscale of the Foot Function Index (FFI). In the study by Macias et al. [15], analyses were conducted in 37 participants in the laser group and 32 in the control group, whereas Gökçe et al. [24] included 30 participants per group. The random-effects meta-analysis showed no statistically significant difference between laser therapy and control interventions in FFI pain scores (SMD = 0.61; 95% CI: −0.91 to 2.13; Z = 0.79; *p* = 0.43). Between-study heterogeneity was extremely high (I^2^ = 94.2%; Tau^2^ = 1.126; χ^2^ = 17.23; df = 1), indicating substantial inconsistency in effect estimates (Figure 5).

Individual study analyses revealed opposing directions of effect. Macias et al. [15] reported a small, non-significant effect favoring laser therapy (SMD = −0.16; 95% CI: −0.62 to 0.31), whereas Gökçe et al. [24] demonstrated a statistically significant effect favoring the control intervention (SMD = 1.39; 95% CI: 0.83 to 1.95), indicating greater pain reduction in participants not receiving laser therapy.

Overall, the available evidence does not support a beneficial effect of laser therapy on pain as measured by the FFI pain subscale in patients with plantar fasciitis. The marked heterogeneity between studies and the small number of available trials highlight the need for further research using standardized pain assessment instruments, harmonized laser treatment protocols, and larger sample sizes to generate more precise and comparable estimates.

Secondary outcomes assessed as baseline–final change are presented in Table 8.

#### 3.3.5. HTI—Heel Tenderness Index (Δ)

Three randomized controlled trials, including a total of 79 participants in the laser intervention groups and 81 in the control groups, evaluated the effect of treatment on heel tenderness using the Heel Tenderness Index (HTI). In the study by Akkurt et al. [17], outcome changes were analyzed in 25 participants in the laser group and 27 in the control group; Ordahan et al. [16] included 35 participants per group, while Zare Bidoki et al. [25] evaluated 19 participants in each study arm.

**Figure 6:**
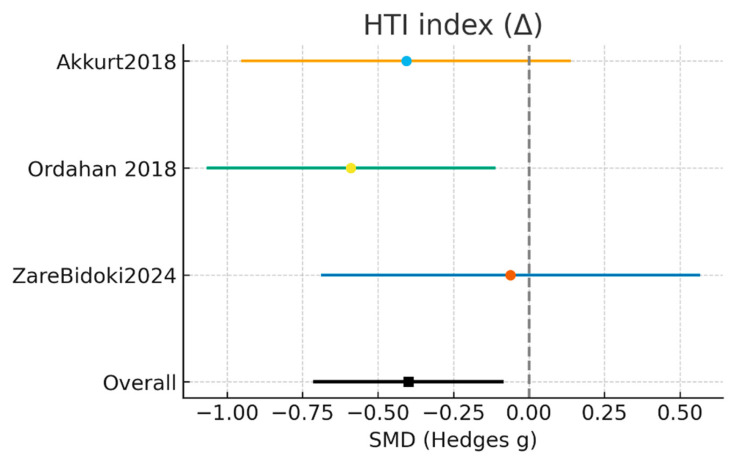
Results of the HTI—Heel Tenderness Index (Δ) meta-analysis [16,17,25].

The random-effects meta-analysis demonstrated a statistically significant reduction in HTI favoring laser therapy (SMD = −0.40; 95% CI: −0.71 to −0.09; Z = −2.53; *p* = 0.011). No between-study heterogeneity was detected (I^2^ = 0%; Tau^2^ = 0.000; χ^2^ = 1.75; df = 2), indicating a high degree of consistency in both the magnitude and direction of the estimated effects across studies (Figure 6).

Individual study analyses showed concordant results. Akkurt et al. [17] reported a favorable, although non-significant, effect for laser therapy (SMD = −0.41; 95% CI: −0.95 to 0.13), whereas Ordahan et al. [16] observed a larger and statistically significant effect (SMD = −0.59; 95% CI: −1.06 to −0.12). Zare Bidoki et al. [25] reported a small and non-significant effect (SMD = −0.06; 95% CI: −0.68 to 0.56), while maintaining the same direction of effect as the other trials.

Taken together, these findings indicate that laser therapy produces a significant reduction in heel tenderness on palpation, as measured by the HTI, compared with control interventions. The absence of heterogeneity supports the robustness of this result. Nevertheless, further randomized trials with larger sample sizes and standardized treatment protocols are required to confirm these findings and to establish their long-term clinical relevance.

Additional secondary outcomes are summarized in Table 9.

#### 3.3.6. Sensitivity Analyses (r = 0.5 and r = 0.8)

Sensitivity analyses were conducted using two alternative assumed correlation coefficients (r = 0.5 and r = 0.8) to assess the robustness of the pooled estimates derived from pre–post change scores (Table 10). Across all evaluated outcomes, the direction and statistical significance of the pooled effects remained unchanged, indicating that the main findings were not sensitive to the choice of correlation coefficient.

The remaining meta-analysis results are presented in Table 10.

For overall pain intensity (VAS) and functional outcomes (FFI total, ASQoL, and SF-36), the pooled effects remained non-significant under both correlation assumptions and were associated with moderate to high heterogeneity. Although minor variations in effect size magnitude and heterogeneity estimates were observed, the absence of statistically significant effects persisted, supporting the robustness of the conclusion that laser therapy does not confer superior improvements in global pain perception or physical function compared with comparator interventions. Similarly, for plantar fascia thickness, sensitivity analyses confirmed the lack of significant between-group differences under both r values, with consistently low heterogeneity. These findings reinforce the conclusion that laser therapy does not produce measurable short-term structural changes detectable by ultrasound.

In contrast, for heel tenderness assessed using the Heel Tenderness Index (HTI), the statistically significant effect favoring laser therapy remained stable across both sensitivity scenarios. Although slight variations in effect size and heterogeneity were noted, the persistence of statistical significance supports the robustness of this outcome and suggests a consistent beneficial effect of laser therapy on localized heel pain elicited by palpation.

Overall, these sensitivity analyses demonstrate that the conclusions of the meta-analysis are methodologically robust, as reasonable variations in the assumed correlation coefficient do not materially affect the pooled estimates or their interpretation.

## 4. Discussion

The findings of this meta-analysis indicate that the clinical efficacy of laser therapy in PF is outcome-dependent and influenced by laser modality and methodological heterogeneity among the included trials. Although a statistically significant reduction in heel tenderness, as assessed by the Heel Tenderness Index (HTI), was observed, no significant effects were found for self-reported pain intensity, physical function, or plantar fascia thickness compared with control interventions. These results should be interpreted in the context of existing evidence and mechanistic hypotheses suggesting that photobiomodulation exerts its effects through modulation of inflammation, oxidative stress, and cellular repair processes [27,28].

The most consistent finding was the improvement in HTI, which demonstrated a statistically significant effect with no detectable heterogeneity. This pattern suggests a specific benefit of laser therapy on localized pain elicited by palpation, potentially mediated by reductions in local inflammatory mediators and modulation of nociceptive thresholds, as supported by experimental photobiomodulation research [28]. Notably, this effect was observed irrespective of laser modality (LLLT or HILT), in agreement with previous clinical studies reporting analgesic effects of both approaches in tendinopathies [2]. These results partially support the hypothesis that laser therapy may induce clinically meaningful nociceptive modulation in patients with PF.

In contrast, analyses of overall pain intensity measured using subjective scales such as the Visual Analog Scale (VAS) did not demonstrate significant between-group differences. The divergence between global pain perception and localized tenderness may be explained by several factors. Plantar fasciitis is a multifactorial condition involving inflammatory, biomechanical, degenerative, and load-related components [29], and nociceptive modulation alone may be insufficient to produce sustained improvements in overall pain perception. Moreover, the very high heterogeneity observed for pain outcomes (I^2^ > 90%) suggests that treatment effects are strongly protocol-dependent, particularly with respect to laser dose, treatment duration, and application frequency—parameters that are well recognized as critical determinants of therapeutic efficacy [12,30].

An important finding of this meta-analysis is the dissociation between the significant improvement observed in heel tenderness, as measured by the Heel Tenderness Index (HTI), and the absence of consistent effects on overall pain intensity assessed using the Visual Analog Scale (VAS). This discrepancy likely reflects fundamental differences between these outcome measures. HTI evaluates localized, examiner-elicited pain sensitivity at the plantar fascia insertion, whereas VAS represents the patient’s global subjective pain experience throughout daily activities.

One possible explanation is that laser therapy primarily modulates local inflammatory processes and peripheral nociceptive sensitivity, leading to reduced pain on palpation, without substantially influencing the biomechanical overload, degenerative changes, or central pain mechanisms that contribute to persistent symptoms in plantar fasciitis. As a result, improvements in localized tenderness may not translate into meaningful reductions in overall perceived pain or functional limitation. This interpretation aligns with the view of plantar fasciitis as a multifactorial condition, in which isolated modulation of local tissue sensitivity may be insufficient to achieve sustained clinical improvement unless combined with interventions addressing mechanical load and functional deficits.

Similarly, the meta-analysis of functional outcomes did not reveal a significant benefit of laser therapy. This finding is consistent with prior evidence indicating that improvements in function are more closely associated with active interventions such as stretching, strengthening exercises, and the use of orthoses [31,32]. Several trials included in this review combined laser therapy with exercise-based programs and reported superior outcomes compared with control interventions, supporting the notion that laser therapy may function as an adjunct rather than a substitute within a multimodal treatment strategy. This interpretation aligns with current clinical guidelines, which recommend active rehabilitation approaches as first-line management for PF [33].

With respect to plantar fascia thickness, the available evidence did not demonstrate clinically meaningful reductions attributable to laser therapy. This observation is consistent with ultrasound-based studies showing that structural changes in the plantar fascia typically occur slowly and may not parallel short-term symptomatic improvement [34]. The absence of detectable morphological effects suggests that the potential benefits of laser therapy are more likely mediated by cellular and neurophysiological mechanisms rather than by rapid tissue remodeling detectable on imaging.

From a clinical perspective, these findings suggest that laser therapy may have a role as part of a comprehensive treatment program, particularly for reducing localized heel tenderness. However, the current evidence does not support its use as a standalone intervention for improving global pain, functional capacity, or plantar fascia structure. This conclusion is consistent with previous systematic reviews reporting limited or inconsistent advantages of laser therapy over other physical modalities such as extracorporeal shock wave therapy or ultrasound [1,4]. Furthermore, the variability in outcomes across trials highlights the importance of adherence to established dosimetric recommendations, such as those proposed by the World Association for Laser Therapy (WALT), as deviations from recommended dose ranges may contribute to suboptimal or inconsistent results [2].

Several limitations of this meta-analysis should be acknowledged. First, there was substantial heterogeneity in laser parameters across the included studies, including wavelength, power output, delivered dose, and treatment protocols. This variability limited the ability to perform robust subgroup or dose–response analyses and may have influenced the pooled effect estimates. Consequently, the results should be interpreted with caution, and future randomized controlled trials with standardized laser protocols and consistent dose reporting are needed to better define optimal treatment parameters. Although laser dose was classified according to WALT recommendations, the limited number of studies per category and incomplete reporting precluded formal subgroup analyses, and no conclusions can be drawn regarding differential effects based on dose adherence.

In particular, the extreme heterogeneity observed in pain-related outcomes suggests that pooling results across highly dissimilar studies may be of limited clinical interpretability. In this context, the absence of consistent direction and magnitude of effect reinforces the conclusion that laser therapy does not demonstrate a reliable or uniform benefit for pain reduction in plantar fasciitis.

In addition, although laser dose was descriptively classified according to World Association for Laser Therapy (WALT) recommendations, formal subgroup analyses based on WALT-adherent versus non-adherent dosing were not feasible due to the small number of studies per outcome and the heterogeneous and often incomplete reporting of dosimetric parameters.

Functional outcomes were assessed using a variety of instruments, including foot-specific scales and generic quality-of-life measures, which capture overlapping but non-identical constructs. Although pooling these outcomes using SMD allowed for a global assessment, this heterogeneity limits the specificity and clinical interpretability of the functional findings. Future studies should prioritize standardized, condition-specific functional measures to improve comparability.

Finally, these findings identify several priorities for future research. Well-designed randomized controlled trials with rigorous standardization of laser dose, energy delivery, application time, and treated areas are needed to reduce methodological variability and clarify true treatment effects. Direct comparisons between LLLT and HILT using dose-equivalent protocols would help determine whether higher-power modalities confer additional clinical benefits. Moreover, studies evaluating laser therapy as an adjunct to active rehabilitation strategies could further elucidate its role within multimodal treatment frameworks. Finally, investigations incorporating inflammatory biomarkers or neurophysiological outcomes may provide valuable insights into the mechanisms underlying the clinical effects of photobiomodulation.

## 5. Conclusions

This systematic review and meta-analysis indicates that in adults with plantar fasciitis, laser therapy may reduce localized heel tenderness, but it does not consistently improve overall pain, physical function, or plantar fascia thickness. These findings suggest that laser therapy may have a limited and symptom-specific effect, likely related to modulation of local tissue sensitivity. Given the substantial heterogeneity across studies and the multifactorial nature of plantar fasciitis, laser therapy should be considered only as an adjunct to established conservative interventions rather than a standalone treatment. Further high-quality randomized trials with standardized protocols are needed to better define its clinical role.

## Figures and Tables

**Table 1:** Characteristics of the included tests.

First Author, Year	Participants at Baseline (Intervention)	Participants at Baseline (Control)	Intervention vs. Control	Outcome and Reassessment Time
Macias et al. [15]	37	32	LLLT (635 nm, 17 mW, 6 sessions/3 weeks) vs. placebo (LED without laser emission)	At 8 weeks: the LLLT group reduced VAS pain by −29.6 ± 24.9 points (44.2%); the placebo group reduced only −5.4 ± 16.0 points (7.5%). Significant between-group difference (*p* < 0.001).
Ordahan et al. [16]	35 (HILT)	35 (LLLT)	HILT vs. LLLT	At 3 weeks: VAS, HTI, FAOS (pain, symptoms, ADL, sports, QoL)—both improve, but HILT shows greater improvement.
Akkurt et al. [17]	25 (HILT + insole)	27 (insole only)	HILT + silicone insole vs. silicone insole alone	At 1 month: both groups improve, but HILT + insole shows greater pain reduction (VAS), larger improvement in HTI and FAOS (pain and QoL) with significant differences (*p* < 0.05).
Sanmak et al. [18]	17	17	ESWT vs. LLLT	Fascial thickness: significant reduction at 1 month in both groups; no between-group differences. FFI: significant improvement at 1 month in both groups; no between-group differences. VAS: significant decrease at 1 month in both groups; no between-group differences.
Rubella et al. [19]	15	15	LLLT vs. Ultrasound	Plantar fascia: minimal and insignificant thickness change (post-2 weeks). Pain: significant reduction in LLLT (*p* = 0.04). Dorsiflexion ROM: significant improvement with ultrasound (*p* = 0.003). FAAM-ADL: significant improvement in LLLT (*p* = 0.05).
Alpturker et al. [20]	20	20	LLLT vs. ESWT	AOFAS: ↑ 63.9 → 75.8; Roles-Maudsley: ↓ 3.0 → 1.85; VAS first steps: ↓ 67.75 → 34.5; VAS exercise: ↓ 78 → 44 (1 month). Within-group differences: AOFAS: 10.65 vs. 11.9; VAS exercise: 26.75 vs. 34. ASQoL, BASFI, BASDAI, MASES improved in both groups. MRI plantar fascia thickness: ↓ 4.43 → 3.66 vs. 4.50 → 3.75 mm.
Naruseviciute & Kubilius [6]	51 (HILT)	51 (LLLT)	HILT vs. LLLT	VAS (various contexts), algometry, ultrasound thickness. Assessments at baseline, 0–3 weeks, 3–4 weeks. Patient-reported subjective efficacy: % with >50% and >75% improvement at 3 weeks.
Güloğlu & Yalçın [21]	32 (LLLT)	35 (ESWT)	LLLT vs. ESWT	VAS: both groups improve; no between-group difference (*p* = 0.876). FFI pain: ESWT superior (*p* = 0.006). FFI disability and activity: both improve, no between-group differences. FFI total: ESWT superior (*p* = 0.033).
Tkocz et al. [22]	30	30	HILT + US vs. Sham HILT + US	VAS pain decreases significantly in both groups from M1 to M4; no between-group differences (*p* > 0.05). LPS decreases in both groups. Follow-up at 1 and 3 months shows slight pain recurrence.
Yetişir et al. [23]	28 (LLLT)	28 (CS)	LLLT vs. CS	VAS palpation: significant within-group improvements (*p* < 0.001). VAS rest: significant only in Group 1. VAS walking/activity: significant in both. FFI: significant in both, greater reduction in CS. HTI: improvements in both groups; timing differs.
Gökçe et al. [24]	30 (LLLT + exercise)	30 (exercise only)	LLLT + exercise vs. exercise	VAS: significant reduction in rest/first step/activity at 1 and 2 months. FFI: significant improvement in pain, disability, total score. AOFAS: significant improvement except alignment; superior to control at both time points.
Zare Bidoki et al. [25]	25 (HILT)	25 (ESWT)	HILT vs. ESWT	VAS: HILT significantly better (*p* = 0.03). HTI: ESWT superior (*p* = 0.006). SF-36, physical and mental health: HILT significantly better.
Ketz et al. [26]	76 (PBMT 10 W + 25 W)	38 (UC)	PBMT vs. Usual Care	6 weeks: pain reduction greater in PBMT; FAAM Sports and ADL improved more in PBMT. At 13 and 26 weeks: PBMT maintains benefit.

ADL: Activities of Daily Living, AOFAS: American Orthopaedic Foot & Ankle Society Score, ASQoL: Ankylosing Spondylitis Quality of Life Questionnaire, BASDAI: Bath Ankylosing Spondylitis Disease Activity Index, BASFI: Bath Ankylosing Spondylitis Functional Index, CS: Control Shoe or Standard Control, ESWT: Extracorporeal Shock Wave Therapy, FAAM: Foot and Ankle Ability Measure, FAOS: Foot and Ankle Outcome Score, FFI: Foot Function Index, HILT: High-Intensity Laser Therapy, HTI: Heel Tenderness Index, LLLT: Low-Level Laser Therapy, MASES: Maastricht Ankylosing Spondylitis Enthesitis Score, MRI: Magnetic Resonance Imaging, PBMT: Photobiomodulation Therapy, QoL: Quality of Life, SF-36: Short Form-36 Health Survey, UC: Usual Care, US: Ultrasound, VAS: Visual Analog Scale.

**Table 2:** LLLT characteristics of the included trials.

First Author, Year	Wavelength (nm)	Average Output Power (mW)	Seconds per Treatment Point (s)	Joules per Point (J)	Number of Treated Spots	Number of Sessions/Weeks	WALT Recommended Dose
Macias et al. [15]	635 nm	17 mW	NR	NR	NR	6 sessions/3 weeks (2 per week)	NR
Ordahan et al. [16] (LLLT)	904 nm	240 mW (superpulsed) *	157.5 s total per session (not defined as individual points)	8.4 J at insertion + 8.4 J at medial border	2 areas treated (insertion + medial fascia)	9 sessions/3 weeks	Not defined by WALT for 904 nm superpulsed in PF
Ordahan et al. [16] (HILT)	1064 nm	8000–12,000 mW (8–12 W)	Phase II: 30 s	Phase I: 150 J; Phase II: 120–150 J	Application over the entire fascia area (continuous circular movements)	9 sessions/3 weeks	No WALT recommendation exists for HILT (Nd:YAG 1064 nm)
Akkurt et al. [17]	1064 nm (Nd:YAG)	Not reported (HILT typically uses 8–12 W, but not stated)	Not applicable (HILT continuous scanning, not point-based)	2000–3000 J per session (total)	Not applicable (continuous scan, not point-based)	10 sessions/3 weeks (alternate days)	No WALT recommendation exists for HILT (Nd:YAG 1064 nm)
Sanmak et al. [18]	685 nm	30 mW	60 s per point (2 points: insertion + along fascia)	2 J/cm^2^ per point	2 treatment zones per session	12 sessions/4 weeks (3/week)	4–6 J per point for soft tissues (no specific PF recommendation for 685 nm)
Rubella et al. [19]	Not specified (visible + infrared)	240	1800 s (30 min at origin) and 300 s (5 min along fascia)	8.4	2 areas: 1.5 cm^2^ at origin, 3 cm^2^ along fascia	12/2	Not reported
Alpturker et al. [20]	830	50	—	8	—	14 sessions/approx. 2 weeks	8 J/cm^2^
Naruseviciute & Kubilius [6]	785	50	6 min 40 s total (phase 1: 1 min; phase 2: 5 min 40 s)	140 total (phase 1: 21; phase 2: 119)	1 scan area over plantar fascia	3/week for 3 weeks (8 sessions)	4 J/cm^2^
Naruseviciute & Kubilius [6]	1064	7000	7 min 8 s total (phase 1: 2 min; phase 2: 5 min 8 s)	3000 total (phase 1: 840; phase 2: 2160)	1 scan area over plantar fascia and heel	3/week for 3 weeks (8 sessions)	120 J/cm^2^
Güloğlu & Yalçın [21]	904	–	60 (5 points × ~12 s each)	8 J/cm^2^	5	15 sessions/3 weeks	8 J/cm^2^
Tkocz et al. [22]	1064	7000	720	4496	1 (applicator covers 30 cm^2^)	5/3 weeks	149.9 J/cm^2^
Yetişir et al. [23]	904	Not specified (only frequency 3500 Hz)	150	2 J/cm^2^	5	10 sessions	As per WALT for plantar fasciitis: standard recommendation 4–8 J/cm^2^ per point
Gökçe et al. [24]	808	1600	30	4	4	10/2 weeks	Not specified
Zare Bidoki et al. [25]	980 ± 10	30 W (30,000 mW)	—	8 J/cm^2^	10 cm^2^ area	9 sessions/3 weeks	—
Zare Bidoki et al. [25]	—	—	Pulse 3000; frequency 12–15 MHz	—	—	9 sessions/3 weeks	—
Ketz et al. [26]	810 + 980 (20%/80%)	10,000 (10 W)/25,000 (25 W)	1 (slow, 10 W)/0.4 (fast, 25 W)	10	Plantar region and calf (serpentine pattern)	3 sessions/week × 3 weeks	10 J/cm^2^

NR: Not Reported. * *p* < 0.05.

**Table 3:** PEDro score.

Study ID	1 *	2	3	4	5	6	7	8	9	10	11	Total	Quality
Macias et al. [15]	+	+	+	+	+	–	+	+	–	+	+	9	High
Ordahan et al. [16]	+	+	+	+	–	–	+	+	–	+	+	7	High
Akkurt et al. [17]	+	+	+	–	+	–	+	+	–	+	+	7	High
Sanmak et al. [18]	+	+	–	+	–	–	+	–	–	+	+	5	Medium
Rubella et al. [19]	+	+	+	+	–	–	+	+	–	+	+	7	High
Alpturker et al. [20]	+	+	+	+	+	–	+	+	–	+	+	8	High
Naruseviciute & Kubilius [6]	+	+	+	+	–	–	+	+	–	+	+	7	High
Güloğlu & Yalçın [21]	+	+	+	+	–	–	+	+	–	+	+	7	High
Tkocz et al. [22]	+	+	+	+	–	–	+	+	–	+	+	7	High
Yetişir et al. [23]	+	+	+	+	–	–	+	+	–	+	+	7	High
Gökçe et al. [24]	+	+	+	+	–	–	+	+	–	+	+	7	High
Zare Bidoki et al. [25]	+	+	+	+	+	–	+	+	–	+	+	8	High
Ketz et al. [26]	+	+	+	+	+	–	+	+	–	+	+	8	High

PEDro Criteria: 1. Specified eligibility criteria; 2. Random assignment; 3. Concealed assignment; 4. Similar baseline groups; 5. Subject blinding; 6. Therapist blinding; 7. Evaluator blinding; 8. Less than 15% dropout rate; 9. Intention-to-treat analysis; 10. Statistical comparisons between groups; 11. Point measures and variability data. * Item not included in the mean score.

**Table 4:** Quality of evidence (GRADE).

Study (Author, Year)	Risk of Bias	Inconsistency	Indirectness	Imprecision	Publication Bias	GRADE Quality
Macias et al. [15]	Moderate	Moderate	Low	Moderate	Low	⬇️ Moderate
Ordahan et al. [16]	Moderate	Low	Low	Moderate	Low	⬇️ Moderate
Akkurt et al. [17]	Moderate	Low	Low	Moderate	Low	⬇️ Moderate
Sanmak et al. [18]	Moderate	Low	Low	High (small sample)	Low	⬇️ Low
Rubella et al. [19]	Moderate	Low	Low	High (very small sample)	Low	⬇️ Low
Alpturker et al. [20]	Moderate	Moderate	Low	Moderate	Low	⬇️ Moderate
Naruseviciute & Kubilius [6]	Moderate	Low	Low	Moderate	Low	⬇️ Moderate
Güloğlu & Yalçın [21]	Moderate	Moderate	Low	Moderate	Low	⬇️ Moderate
Tkocz et al. [22]	Moderate	High	Low	Moderate	Low	⬇️ Moderate
Yetişir et al. [23]	Moderate	Moderate	Low	Moderate	Low	⬇️ Moderate
Gökçe et al. [24]	Moderate	High	Low	Moderate	Low	⬇️ Moderate
Zare Bidoki et al. [25]	Moderate	Moderate	Low	Moderate	Low	⬇️ Moderate
Ketz et al. [26]	Moderate	Low	Low	Moderate	Low	⬇️ Moderate

**Table 5:** Meta-analysis results for pain intensity (baseline–final change).

Study	Mean Int (Δ)	SD Int	N Int	Mean Ctrl (Δ)	SD Ctrl	N Ctrl	Weight %	SMD (g)	95% CI
Macias et al. [15]	−29.6	24.19	37	−5.3	15.99	32	20.2%	−1.15	[−1.66, −0.65]
Akkurt et al. [17]	−4.2	2.1	25	−2.3	1.74	27	19.9%	−0.97	[−1.54, −0.40]
Tkocz et al. [22]	−3.5	1.45	30	−3.0	2.0	30	20.3%	−0.28	[−0.78, 0.22]
Gökçe et al. [24]	−0.5	1.41	30	−3.1	2.36	30	20.0%	1.32	[0.76, 1.87]
Zare Bidoki et al. [25]	−5.69	1.54	19	−6.0	1.58	19	19.6%	0.19	[−0.43, 0.82]
Total	-	-	141	-	-	138	100.0%	−0.18	[−1.06, 0.69]

Heterogeneity: Tau^2^ = 0.920; Chi^2^ = 51.138, df = 4; I^2^ = 92.2%, Test for overall effect: Z = −0.41 (*p* = 0.6821), Random-effects SMD = −0.183 (95% CI −1.060 to 0.693).

**Table 6:** Meta-analysis results for functional outcome (baseline–final change).

Study	Mean Int (Δ)	SD Int	N Int	Mean Ctrl (Δ)	SD Ctrl	N Ctrl	Weight %	SMD (g)	95% CI
Macias et al. [15]	−29.9	39.74	37	−24.7	38.91	32	28.5%	−0.13	[−0.60, 0.34]
Gökçe et al. [24]	−0.2	0.95	30	−1.7	2.55	30	26.3%	0.77	[0.25, 1.29]
Alpturker et al. [20]	−3.2	2.66	20	−3.25	2.47	20	22.9%	0.02	[−0.59, 0.63]
Zare Bidoki et al. [25]	−12.16	18.21	19	−14.63	18.35	19	22.3%	0.13	[−0.49, 0.76]
Total	-	-	106	-	-	101	100.0%	0.2	[−0.22, 0.62]

Heterogeneity: Tau^2^ = 0.102; Chi^2^ = 6.916, df = 3; I^2^ = 56.6%, Test for overall effect: Z = 0.94 (*p* = 0.3498), Random-effects SMD = 0.199 (95% CI −0.218 to 0.617).

**Table 7:** Meta-analysis results for post-treatment pain intensity.

Study	Mean Int (Post)	SD Int	N Int	Mean Ctrl (post)	SD Ctrl	N Ctrl	Weight %	MD	95% CI
Macias et al. [15]	5.6	1.3	37	5.8	1.0	32	8.2%	−0.2	[−0.74, 0.34]
Rubella et al. [19]	2.34	0.23	30	2.52	0.47	30	69.1%	−0.18	[−0.37, 0.01]
Alpturker et al. [20]	3.66	0.61	20	3.75	0.42	20	22.7%	−0.09	[−0.42, 0.24]

Heterogeneity: Tau^2^ = 0.007; Chi^2^ = 1.092, df = 1; I^2^ = 8.4%, Test for overall effect: Z = −1.40 (*p* = 0.1617), Random-effects SMD = −0.280 (95% CI −0.672 to 0.112).

**Table 8:** Meta-analysis results for secondary outcomes (baseline–final change).

Study	Mean Int (Δ)	SD Int	N Int	Mean Ctrl (Δ)	SD Ctrl	N Ctrl	Weight %	SMD (g)	95% CI
Macias et al. [15]	−15.1	19.19	37	−12.2	17.59	32	50.5%	−0.16	[−0.62, 0.31]
Gökçe et al. [24]	−0.1	1.25	30	−2.8	2.4	30	49.5%	1.39	[0.83, 1.95]
Total	-	-	67	-	-	62	100.0%	0.61	[−0.91, 2.13]

Heterogeneity: Tau^2^ = 1.126; Chi^2^ = 17.230, df = 1; I^2^ = 94.2%, Test for overall effect: Z = 0.79 (*p* = 0.4301), Random-effects SMD = 0.610 (95% CI −0.905 to 2.125).

**Table 9:** Meta-analysis results for secondary outcomes (baseline–final change).

Study	Mean Int (Δ)	SD Int	N Int	Mean Ctrl (Δ)	SD Ctrl	N Ctrl	Weight %	SMD (g)	95% CI
Akkurt et al. [17]	−1.2	0.67	25	−0.85	0.99	27	32.6%	−0.41	[−0.95, 0.13]
Ordahan et al. [16]	−1.68	0.77	35	−1.13	1.05	35	42.7%	−0.59	[−1.06, −0.12]
Zare Bidoki et al. [25]	−2.31	0.77	19	−2.26	0.82	19	24.7%	−0.06	[−0.68, 0.56]
Total	-	-	79	-	-	81	100.0%	−0.4	[−0.71, −0.09]

Heterogeneity: Tau^2^ = 0.000; Chi^2^ = 1.751, df = 2; I^2^ = 0.0%, Test for overall effect: Z = −2.53 (*p* = 0.0114), Random-effects SMD = −0.399 (95% CI −0.709 to −0.090).

**Table 10:** Sensitivity analyses (r = 0.5 and r = 0.8).

Outcome	r	Effect Size (95% CI)	Tau^2^	I^2^	Z (*p*)
Pain (VAS)	0.5	−0.18 [−1.09, 0.73]	0.94	92.0%	−0.39 (*p* = 0.70)
Function	0.5	0.21 [−0.23, 0.64]	0.10	56.0%	0.93 (*p* = 0.35)
Fascia thickness	0.5	−0.17 [−0.65, 0.12]	0.01	8.0%	−1.36 (*p* = 0.17)
HTI	0.5	−0.40 [−0.71, −0.09]	0.00	0.0%	−2.53 (*p* = 0.011)
Pain (VAS)	0.8	−0.20 [−1.01, 0.62]	0.81	89.5%	−0.48 (*p* = 0.63)
Function	0.8	0.24 [−0.18, 0.66]	0.12	59.0%	1.10 (*p* = 0.27)
Fascia thickness	0.8	−0.19 [−0.68, 0.11]	0.01	9.0%	−1.41 (*p* = 0.16)
HTI	0.8	−0.43 [−0.75, −0.11]	0.01	7.0%	−2.62 (*p* = 0.009)

## Data Availability

Data supporting the findings of this study are available within the article and its Supplementary Materials. No new datasets were generated or analyzed.

## Supplementary Materials

The following supporting information can be downloaded at: https://www.mdpi.com/article/10.3390/jcm15031307/s1, PRISMA 2020 checklist.
